# Implementation of the 2018 Classification of Periodontal Diseases: A Questionnaire-Based Survey

**DOI:** 10.1055/s-0045-1806951

**Published:** 2025-04-23

**Authors:** Sofia Zarenti, Aikaterini Elisavet Doufexi, Leonidas Batas, Domniki Chatzopoulou, David Gillam, Nikos Mardas, Dimitra Sakellari

**Affiliations:** 1 Department of Preventive Dentistry, Periodontology and Implant Biology, Dental School, Aristotle University of Thessaloniki, Thessaloniki, Greece; 2 Center Immuno-Biology & Regenerative Medicine, Institute of Dentistry, Barts and The London School of Medicine and Dentistry, Queen Mary University of London, London, United Kingdom; 3 Centre for Oral Bioengineering, Institute of Dentistry, Barts and The London School of Medicine and Dentistry, Queen Mary University of London, London, United Kingdom; 4 Centre for Oral Clinical Research, Barts and The London School of Dentistry, London, United Kingdom

**Keywords:** classification, periodontal diseases, questionnaire, diagnosis

## Abstract

**Objectives:**

The new classification of periodontal and peri-implant diseases and conditions (2018) includes definitions of the respective diseases, modifications, and new additions to the 1999 classification. The purpose of the study was to present the opinion and level of understanding of Greek dentists regarding the new classification and to determine the approach for its establishment.

**Materials and Methods:**

A questionnaire consisting of 26 multiple-choice questions was distributed to Greek dentists in paper or digital form.

**Statistical Analysis:**

Data were collected, recorded using Microsoft Excel, and were analyzed with SPSS 29.0 (IBM, United States).

**Results:**

A total of 203 questionnaires were collected (49.3% male/50.2% female; mean age 39.02 years). 36.8% of the participants exclusively practiced periodontology and 44.8% general dentistry. Seventy-one participants were members of the Hellenic Society of Periodontology (HSP). 58.9% used the old classification system, which is reported as preferred by 53.5% of HSP members. Also, 53.3% of participants characterized the new classification as difficult to be applied, 47.1% of whom were HSP members. The most frequent reason for not using it (63/133) was the convenience of the older system application. A statistically significant higher percentage of HSP members correctly answered the comprehension questions (diagnostic criteria and clinical cases) of the questionnaire ( *z* -test with Bonferroni correction < 0.05). Note that 79.7% of the participants stated that they should improve their knowledge regarding the new classification, mainly through seminars/courses.

**Conclusion:**

Most Greek dentists appear not to implement the 2018 classification system due to reported difficulty in understanding, while recognizing the need to comprehend and apply it to their clinical practice.

## Introduction

Classification systems contribute not only to determine etiology, diagnosis, and treatment planning of various diseases, but also to improve and establish effective communication between dentists, either general or specialists.

In November 2017, the World Workshop on the Classification of Periodontal and Peri-implant Diseases and Conditions was held, cosponsored by the American Academy of Periodontology (AAP) and the European Federation of Periodontology (EFP), which included expert participants from all over the world. In March 2018, the scientific articles on the new classification of periodontal and peri-implant diseases were published. Moreover, in May 2020 and June 2022, clinical guidelines for treatment of stage I - III and IV periodontitis, respectively, based on the new classification (2018), were also published. 1 2 The 2018 classification includes the definitions of periodontal and peri-implant diseases, criteria for their diagnosis based on specific characteristics, and the differences compared to the 1999 classification of periodontal diseases and conditions. 3

According to the 2018 classification, periodontitis is not characterized as chronic or aggressive. Instead, the disease is defined based on a multidimensional staging and grading system. 4 The stage of periodontal disease is based on the measurable amount of destroyed periodontal tissues (clinical attachment loss, radiographic bone loss, and tooth loss attributed to periodontitis) and all the specific factors that define the complexity of the indicated periodontal and rehabilitation treatment. The grade is an indicator of the rate of periodontitis progression, responsiveness to standard therapy, and potential impact on systemic health and is based on direct evidence of radiographic bone loss between two different time points, 5 years apart, or radiographic bone loss in relation to patient age (% bone loss/age). The grade can be further modified with the presence of risk factors such as smoking or uncontrolled diabetes. 5 The extent and distribution of periodontal disease is similarly to the previous classification described as localized (< 30% of teeth involved), generalized, or molar/incisor pattern.

In a study by Ravidà et al, specialist periodontists categorized 10 periodontitis cases using the staging and grading of the new classification system. It was concluded that there was a moderate interexaminer consistency. 6 However, Abrahamian et al reported that using the new classification system resulted in high interexaminer consistency, regardless of the experience of examiners. 7

It is still questionable to what extent the new classification system has been adopted and implemented in everyday practice by dental clinicians.

The purpose of this study was to (1) evaluate demographic information and the opinion of professionals (general dental practitioners or specialists periodontists) regarding the new classification system of periodontal diseases, (2) to investigate the level of understanding of the 2018 classification system, and (3) to determine the educational needs aiming at the better dissemination and application of the new classification in clinical practice.

## Materials and Methods

### Study Design and Questionnaire

A 26-item questionnaire that included multiple-choice questions on the new classification of periodontal diseases was distributed to a convenience sample of Greek dentists. The participants were either general dental practitioners, dental specialists, or postgraduate dental students. The questionnaires were physically distributed within the Dental School Aristotle University of Thessaloniki, Greece, and in two national conferences (Congress of Periodontology and Congress of General Dentists) between March 2023 and August 2023.

The questionnaire was written in both Greek and English languages and consisted of three parts. The purpose of the first part was to collect demographic information of participants and to examine the opinion of professionals regarding the 1999 and 2018 classification. The second part included generic and clinical case-based questions to assess the understanding of the new classification system. The final part aimed to gain insight into the necessary changes for better integration of the 2018 classification system in every day clinical practice. The questions were either multiple-choice questions or 10-point scales. The questionnaire is presented as Supplementary Material .

### Ethical Considerations

The study was approved by the Ethical Committee of the School of Dentistry, Aristotle University of Thessaloniki, Thessaloniki, Greece (# 185/20-02-2023).

### “Gold Standard” Answers

Regarding the generic and clinical case-based questions of the second part of the questionnaire, the correct answers were determined by four experienced periodontists among the personnel of the department according to the official proceedings of the *Journal of Periodontology* , after discussion in case of subsequent disagreement.

### Data Analysis

Data were collected, recorded using Microsoft Excel, and were analyzed with SPSS 29.0 (IBM, United States).

## Results

### Demographic Information and Dental Professionals' Opinion

All the 203 dentists that were invited to participate in this survey returned the questionnaires (100 males; 102 females [1 missing value], 49.3%/50.2%, mean age 39.02 ± 12.08 years). Ninety (44.8%) participants were general practitioners (GPs). Seventy-four participants (36.8%) had a practice limited to one dental specialty; periodontology (SP) ( *n* = 50), implantology ( *n* = 10), prosthodontics ( *n* = 3), orthodontics ( *n* = 2), endodontics ( *n* = 2), oral medicine ( *n* = 2), and pediatric dentistry ( *n* = 1). The rest of the participants ( *n* = 37) practiced more than one dental specialties (2 missing values). The mean years since graduation were 15.36 years (standard deviation: 11.71) (10 missing values). Most of the responders (57.5%) practiced dentistry in a large urban center, 51 (25.5%) in an urban center, 29 (14.5%) at a local region, and 5 (2.5%) in more than one place (3 missing values).

Among the 203 responders, 65 (32%) were subscribed to at least one dental journal (68.8% had one or two subscriptions). Seventy-one (35.1%) of the participants were members of the Hellenic Society of Periodontology (HSP) (1 missing value).

Τhe majority of dentists (79.7%) claimed to diagnose periodontitis in patients 0 to 5 times a day. Among the participants ( *n* = 41) who diagnose periodontitis in patients more than five times a day, 70.7% ( *n* = 29) were members of the HSP ( *z* -test with Bonferroni correction < 0.05).

One hundred seventy-nine participants (89.5%) (3 missing values) rated their interest in the classification of periodontal disease between 5 and 10 on a 10-point scale.

**Fig. 1:**
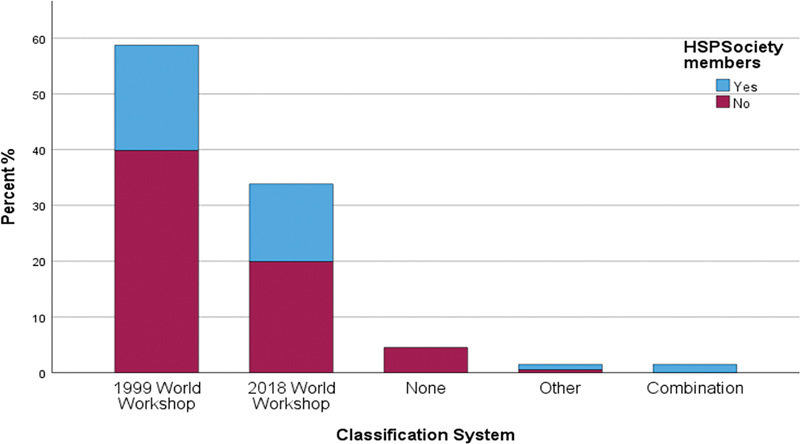
Classification systems used by participants (% distribution).

**Fig. 2:**
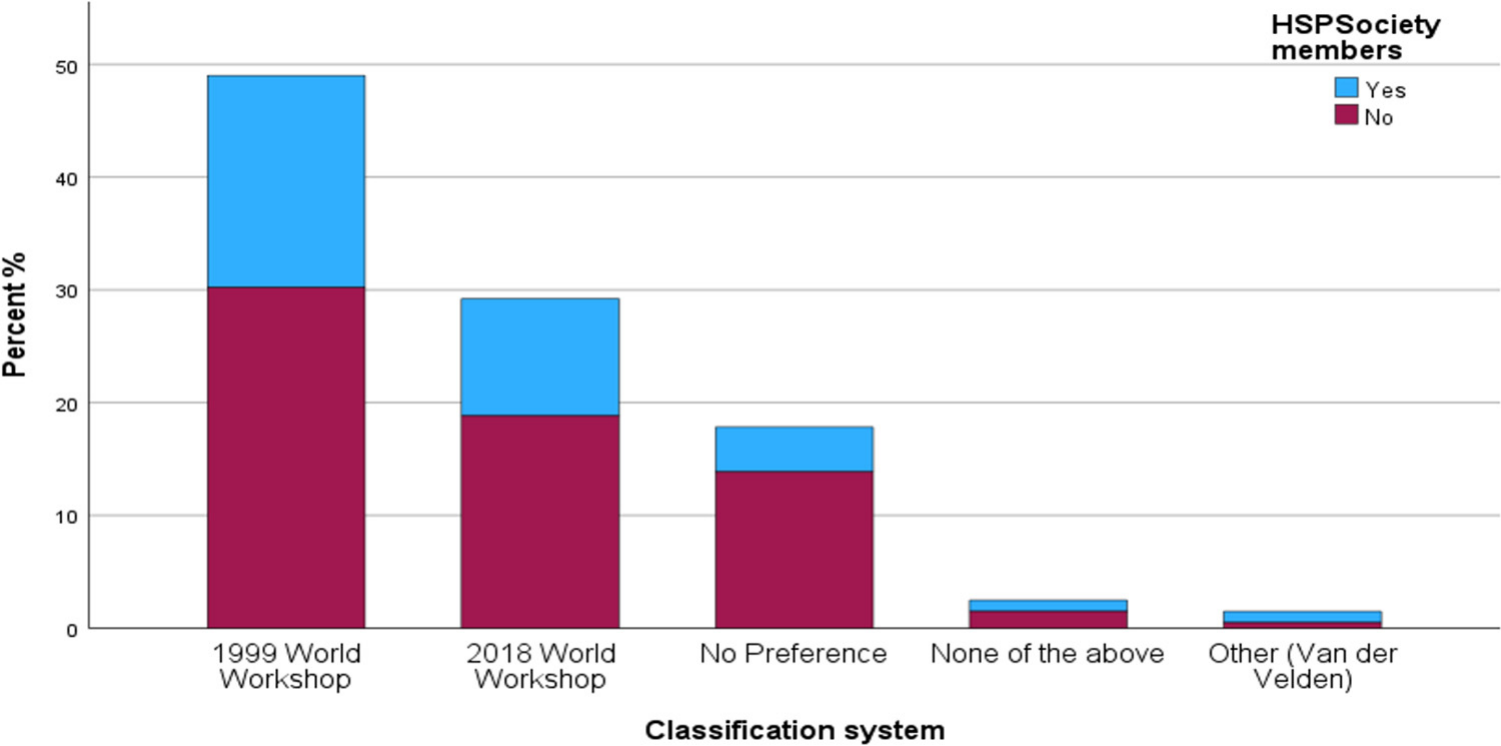
Preference of classification systems in percentages.

Note that 58.9% of participants stated that they used the previous classification system (1999), as did 53.5% of HSP members. Sixty-eight participants (33.7%) used the new classification system (2018), as did 28 (39.4%) HSP members ( Fig. 1 ). Regarding preference, 99 responders (49%)—53.5% of HSP members—preferred the previous classification of periodontal disease. Fifty-nine participants (29.2%)—29.6% of HSP members—preferred the new classification ( Fig. 2 ).

In the two 10-point scales of questionnaire the participants indicated how confident they were using the 1999 and the 2018 classification system, respectively. Note that “1” meant not confident at all and “10” highly confident. Forty-one percent of non-HSP members and 71.4% of HSP members responded “8 or more” regarding the old classification system use. Note that 32.5% of non-HSP members and 37.1% of HSP members answered “8 or more” regarding the new classification system use.

**Fig. 3:**
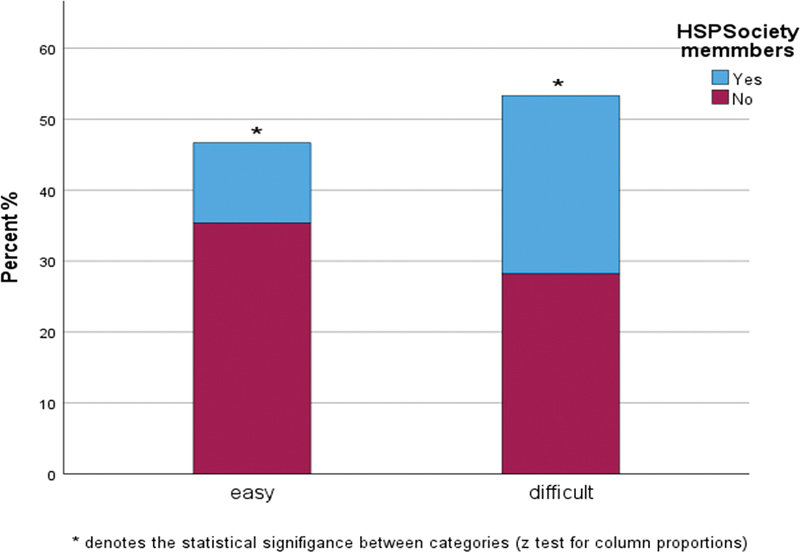
Ease of use of the 2018 classification system (as reported).

When asked how difficult the new classification system was in practice, 53.3% of dental professionals indicated that the implementation of the 2018 classification was difficult, and so did 69% ( *n* = 49) of the HSP members ( Fig. 3 ).

Regarding the way/ways dentists acquired their awareness and knowledge of the new classification system, the three most frequent responses were: “I read the official proceedings from the Journal of Periodontology (JOP)/ Journal of Clinical Periodontology (JCP)”—95 responses, “Continuing Professional Development courses/lectures/congresses-66 responses, and “peer discussion”—44 responses.

To the follow-up question about the reason of not using the new classification system, most dentists (67/132, 50.7%) answered that they were more comfortable using a different classification system ( Table 1 ).

**Table 1:** Reasons for not using the 2018 classification system

Reason not using (percentage %)	HSP members		In total (%)
	Yes	No	
I do not understand it	7.0	2.2	3.8
I feel more comfortable using a different classification system	51.2	44.9	47.0
It is a very time-consuming process	23.3	19.1	20.5
I do not know/aware of a new classification	0.0	24.7	16.7
Other	9.3	5.6	6.8
Takes too long and unaware of a new classification	2.3	0.0	0.8
I feel more comfortable using a different classification system takes too long	4.7	1.1	2.3
I feel more comfortable using a different classification system, difficult for a general dentist	2.3	0.0	0.8
I do not always have an OPG/X-ray machine	0.0	1.1	0.8
I feel more comfortable using a different classification, takes too long	0.0	1.1	0.8

Abbreviations: HSP, Hellenic Society of Periodontology; OPG, orthopantomogram.

When asked for how long have they used the new classification system, 22/107 (20.6%) dentists indicated 6 months, 13/107 (12.1%) said 6 to 9 months, and 72/107 (67.3%) replied 1 year or more. Most HSP members (35/45, 77.8%), however, responded 1 year or more. Referring to the average time needed to clinically classify periodontal patients, 27.6% ( *n* = 32) stated less than 3 minutes, 37.9% ( *n* = 44) 4 to 5 minutes, 17.2% ( *n* = 20) said 6 minutes, 13.8% ( *n* = 16) 10 to 14 minutes, and 3.4% ( *n* = 4) stated 10 to 15 minutes. Among the HSP members, the most frequent response in this question was less than 3 minutes, which differed significantly from the non-HSP members category ( *z* -test with Bonferroni correction < 0.05).

### Comprehension Questions Regarding the New Classification

Regarding the extent and distribution of periodontitis, 46.9% ( *n* = 90) (11 missing values) of all participants and 63.2% ( *n* = 44) (3 missing values) of HSP members agreed with the “gold standard” answer of “> 30% teeth affected” ( *z* -test with Bonferroni correction < 0.05). When asked about the primary characteristic of staging, 33.3% ( *n* = 64) (11 missing values) of all participants and 55.2% ( *n* = 37) (4 missing values) of HSP members agreed with the “gold standard” answer of “the interdental clinical attachment loss (at the site of greatest loss)” ( *z* -test with Bonferroni correction < 0.05). For the follow-up question on the parameters used for grading in case the dentist did not have any previous radiographic data for the patient, the agreement rate with the “gold standard” answer was 33.5% for all participants ( *n* = 65) (9 missing values) and 44.1% for HSP members ( *n* = 30) (3 missing values) ( *z* -test with Bonferroni correction < 0.05).

**Fig. 4:**
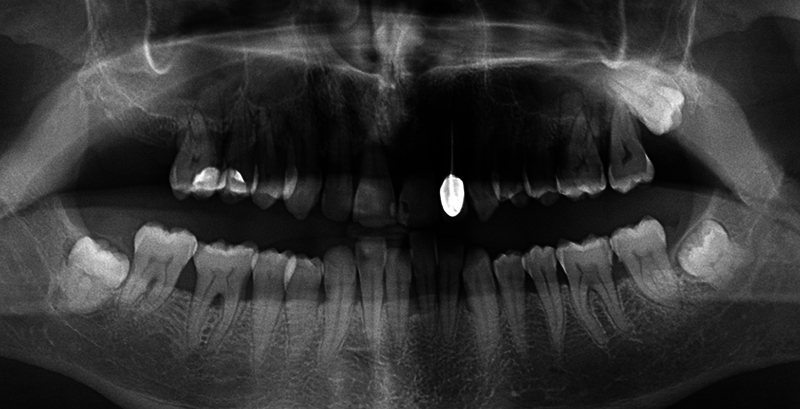
Clinical case no 1-Panoramic X-ray.

**Fig. 5:**
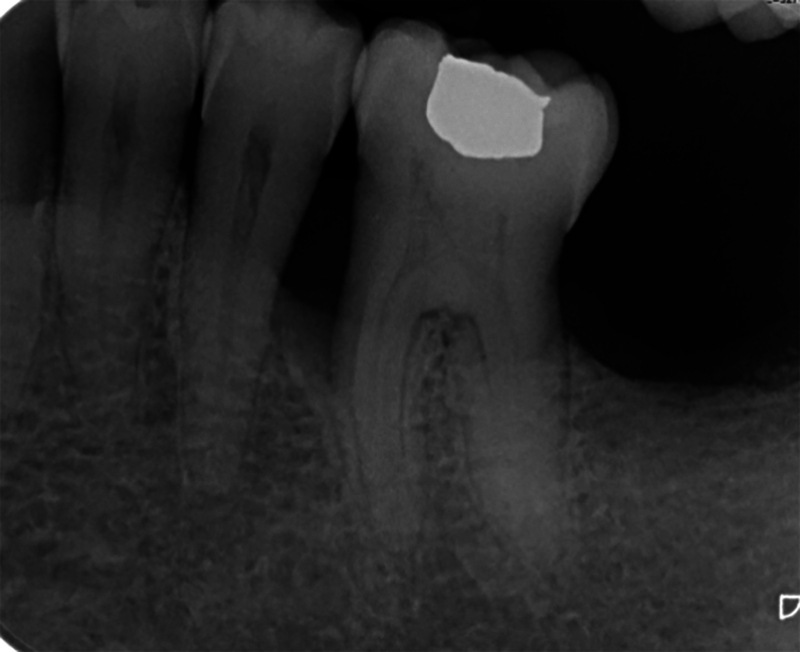
Clinical case no 2—Intraoral X-ray.

Based on a panoramic ( Fig. 4 ) and an intraoral X-ray ( Fig. 5 ), dentists were asked to categorize the clinical cases by stage and grade, respectively. Gold standard answers were “stage III” and “grade C.” Note that 56.7% ( *n* = 110) (9 missing values) of all dental professionals and 67.6% of HPS members ( *n* = 46) (3 missing values) agreed with the “gold standard” answer for staging ( *z* -test with Bonferroni correction < 0.05) while 49.2% ( *n* = 95) (10 missing values) of all participants and 63.2% ( *n* = 43) (3 missing values) of HPS members agreed with the “gold standard” answer grading ( *z* -test with Bonferroni correction < 0.05).

### Future Development

One hundred fifty-seven dentists (79.7%) (5 missing values) stated that they needed to improve their knowledge and understanding regarding the 2018 classification. With respect to knowledge improvement, most dentists (88/178, 49.4%) preferred through Continuing Education Courses. Webinars (45/178, 25.3%) and journals (36/178, 20.2%) were among the most frequent options.

## Discussion

Classification systems should facilitate accurate and reproducible diagnosis to avoid miscommunication between clinicians and promote appropriate customized treatment for the patients. However, the adoption and implementation of any new classification and diagnostic system is always time consuming. 8

This study aimed to assess the perception, comprehension, and implementation of the 2018 classification system of periodontal disease among dentists practicing in Greece. The results revealed that only 33.7% of responders were using this classification system, and even fewer (29.2%) preferred it over the previous classification systems. The majority of dentists who did not use it were more confident diagnosing periodontal disease based on the 1999 classification. Nevertheless, 46.7% of participants reported that the new classification system was user-friendly whereas surprisingly 69% of the HSP members found it difficult. This observation may suggest that GPs might be less meticulous in diagnosing periodontal disease patients, whereas the correct identification of the periodontal severity and complexity of its treatment would enable them to refer complicated cases to specialists. As a result, they may not apply the new classification system in every clinical situation involving periodontally compromised patients. On the other hand, it seems that HSP members, although they are knowledgeable about the most recent classification system, are reluctant to apply it in everyday practice due to its complexity. Since the 2018 classification system is based on a multilevel definition of staging and grading of the disease taking into consideration various systemic risk factors (diabetes, smoking) and clinical parameters, it may be considered somewhat complicated, more time consuming, and less user-friendly for the everyday clinical practice compared to the previous one.

To evaluate comprehension and agreement with the gold standard, the questionnaire included diagnostic criteria. Interestingly, the highest level of agreement (46.9%) was achieved when the diagnostic criterion was identical to the old classification system (regarding the extent of the disease). Both in diagnostic criteria and clinical cases, HSP members showed statistically significantly higher agreement to the “gold standard answers” as set by specialists than non-members. It should also be taken into consideration that HSP members diagnosed periodontitis more frequently and in less time than non-members which would reinforce the members' assertion of the difficulties involved when using the new classification system.

Recent studies have explored various aspects of the implementation and reliability of the 2018 classification system. In a recent study by Ravidà et al, specialists periodontists utilized the 2018 new classification system to categorize 10 clinical cases of periodontitis. 6 The results indicated moderate interexaminer consistency for stage ( *K* value: 0.49), grade ( *K* value: 0.50), and extent ( *K* value: 0.51). One year later, Abrahamian et al conducted a survey in which five clinical cases of periodontitis were included. Authors examined the interexaminer agreement among faculty, graduates, and students from 89 postgraduate programs and reported a high interexaminer consistency (68.7% for stage, 82.4% for grade, and 75.5% for extent), regardless of the experience level of the examiners. 7 However, in the present study, only 39.3% of participants were specialized in periodontology and may therefore be one of the reasons why the rates of agreement with the “gold standard answers” were notably lower. Nevertheless, an observation focused solely on HSP members' responses revealed a higher percentage of agreement ranging from 44.1 to 67.6%, indicating moderate agreement. Similarly, in a study designed to evaluate the agreement between examiners with different levels of education and expertise when assigning the 2018 classification case definitions to dental implants, the results revealed moderate consistency. 9 Another study assessing interexaminer reliability among undergraduate students found high consistency in diagnosing periodontitis using the 2018 classification system, underscoring its applicability in educational settings. 10 While the 2018 classification system introduces a comprehensive framework for staging and grading, its complexity has posed challenges in clinical practice. For example, some studies have highlighted difficulties in consistent implementation across diverse clinical settings due to the intricate nature of the system. 11 These findings suggest a need for simplification or additional resources to support its widespread adoption.

Frameworks such as Assessment, Classification, Etiology, and Severity have been developed to facilitate the application of the new classification system in clinical practice. 12

In this study, it is suggested that the new classification system is complicated, but dentists and specialists are eager to be more involved in diagnosing periodontal cases based on both the new (2018) and the old (1999) classification system. Furthermore, the study's results are based on answers of both GP and SP, which is an advantage, and using these results as a pilot study, in the future it could be very informative to include dentists' and specialists' opinions who have variant geographical and academic backgrounds.

This study is limited by its relatively modest sample size, which may affect the generalizability of the findings. Future research should aim to include larger and more diverse cohorts to enhance the reliability of the results. Additionally, the uneven distribution of participants across specialties limits the representativeness of the findings. Ensuring equal representation of dental specialties in future studies will provide a more comprehensive understanding of the adoption and challenges associated with the 2018 classification system. One limitation of this study is the absence of detailed geographic analysis of participants' responses. While the study captured broad urban and nonurban distinctions, regional differences were not explored. Geographic variations in access to training and resources may influence the adoption of the 2018 classification system. Future research should stratify responses by region or city to identify localized barriers and facilitators, enabling targeted educational and resource-based interventions. Another limitation is its reliance solely on closed-ended questions, which, while effective for quantitative data collection, restricts the ability to explore nuanced opinions, motivations, and experiences of participants. Future studies should incorporate open-ended questions to provide richer qualitative insights, such as identifying specific challenges in adopting the 2018 classification system or recommendations for improving its integration into clinical practice. A mixed-methods approach could complement the current findings and offer a deeper understanding of practitioners' perspectives.

Since 2018, both the University of Athens and Aristotle University of Thessaloniki have incorporated the new classification system of periodontal disease and conditions into their dentistry curriculums. Α study at the International University for Science and Technology in Syria demonstrated positive student feedback and effective clinical application when the system was integrated into the curriculum. 13 It is forecasted that in the upcoming years, more dentists will adopt and prefer this classification system following targeted teaching of the classification and its transition to clinical practice. Seminars and courses organized by the dental societies and academies, as suggested by answers of the present questionnaires, will also improve the awareness and implementation of the 2018 classification.

Future research should expand on these findings by conducting longitudinal studies to track changes in adoption over time and exploring the impact of targeted educational programs. Additionally, analyzing interregional variations and collecting qualitative data through interviews or open-ended survey questions could provide a more holistic understanding of the challenges associated with the new classification system.

## Conclusion

Classification of each case of periodontitis is important for diagnosis and a personalized treatment plan. Therefore, the aim of this study was to investigate the use of the 2018 classification system by periodontists and GPs and compare it with the advantages and disadvantages of the 1999 classification system. The present study reveals a significant reluctance of Greek dentists to implement the 2018 classification system, with almost 60% of practitioners still relying on the 1999 system. The main reason for not using was the difficulty of understanding. Despite the resistance, there is a clear recognition of the need for further education aimed at establishing it in clinical practice. Moreover, this investigation implies the need for an easier system in a future classification.
