# Supplementary material for: Implementation of the 2018 Classification of Periodontal Diseases: A Questionnaire-Based Survey
Source: Eur J Dent. 2025 Apr 23;20(1):188–94. doi: 10.1055/s-0045-1806951 (PMC12890400; doi:10.1055/s-0045-1806951)
Supplement: Supplementary file 1 — Supplementary Material [file 10-1055-s-0045-1806951-s24123754.pdf]

Periodontal Classification Questionnaire

Dear Colleague,

We are very interested in your opinions regarding the use of the new 2017 World Workshop (WWP) classification of periodontal diseases in your day-to-day clinical practice.

The questionnaire is divided into three parts; Part 1 aims to collect information regarding demographics and the profession's opinions of the systems used to classify periodontitis (i.e. 1999 World Workshop classification, 2017 World Workshop Classification and 2019 British Society of Periodontology implementation), Part 2 aims to investigate the current level of understanding of the new 2017 World Workshop (WWP) classification system and Part 3 evaluates any future development needs regarding the use of the new periodontal classification system. Your replies will be confidential and the data will be anonymized in order to provide information on how the new classification/implementation systems are currently being used in general practice.

We would be grateful if you could complete this questionnaire. It should only take a few minutes of your time and it will benefit our current research which is in collaboration between Barts and The London School of Medicine and Dentistry, Queen Mary University of London and the Department of Preventive Dentistry, Periodontology and Implant Biology of the Aristotle University of Thessaloniki.

Thank you for your help,

PART 1

This section aims to evaluate your opinion of the systems used to classify periodontitis.

1. **Year of Birth:**

2. **Gender:** ☐ Male ☐ Female

3. **Type of practice:**

   ☐ Periodontology ☐ General Dentistry ☐ Implant

   ☐ Other (Please Specify)……………………………

4. **Year of Graduation:** [ ]

5. **Where do you practice dentistry?**

   ☐ In a large urban center ☐ In an urban center ☐ In the region

6. A) Do you subscribe to any Periodontology Journals?

☐ Yes ☐ No

B) If your answer is "yes", Please provide how many journals you have subscribed to? ☐

7. Are you a member of Hellenic Society of Periodontology?

☐ Yes ☐ No

8. How frequently do you need to diagnose periodontitis in patients on a daily basis? (Please tick one)
   - ☐ 0-5 times a day
   - ☐ 5-10 times/day
   - ☐ More than 10 times/day

9. How would you rate your interest in the classification of periodontal disease? (Please circle)

(Low) 0 1 2 3 4 5 6 7 8 9 10 (High)

10. What system are you most commonly using to classify periodontitis in patients? (Please tick one)
   - ☐ 1999 World Workshop (Chronic/Aggressive)
   - ☐ 2017 World Workshop (Stage/Grade)
   - ☐ None of the above
   - ☐ Other (Please specify)……………………………………………………………………………….

11. Which classification/implementation system do you prefer? (Please tick one)
   - ☐ 1999 World Workshop (Chronic/Aggressive)
   - ☐ 2017 World Workshop (Stage/Grade)
   - ☐ No preference
   - ☐ None of the above
   - ☐ Other (Please specify)……………………………………………………………………………….

12. How confident did you/do you feel in using the 1999 World Workshop (chronic/aggressive) classification system? (Please circle)

(Low) 0 1 2 3 4 5 6 7 8 9 10 (High)

13. How confident do you feel in using the 2017 World Workshop (stage/grade) classification system? (Please circle)

(Low) 0 1 2 3 4 5 6 7 8 9 10 (High)

14. **How would you assess the ease of the 2017 World Workshop (stage/grade) classification system? (Please circle)**
    - ☐ Too easy
    - ☐ Quite easy
    - ☐ Easy
    - ☐ Difficult
    - ☐ Quite difficult
    - ☐ Too difficult

15. **How have you learnt about the 2017 World Workshop classification system? (Please tick one or more)**
    - ☐ Reading official proceedings from the Journal of Periodontology
    - ☐ Reading other journals
    - ☐ Reading newsletters
    - ☐ Online Webinars
    - ☐ Peer discussion
    - ☐ Continual Professional Development courses/lectures/congresses
    - ☐ Industry representatives
    - ☐ Social Media
    - ☐ Not learnt yet
    - ☐ Other (please specify) ……………………………………………………………………………………..

16. **If you are not using the new classification system, what is the reason for this? (Please tick one or more)**
    - ☐ I do not understand it
    - ☐ I feel more comfortable using a different classification system
    - ☐ It takes too long
    - ☐ I am not aware of a new classification system
    - ☐ Other (please specify) ……………………………………

17. **If you are using the new classification, for how long have you been using it? (Please tick one)**
    - ☐ Less than a few months
    - ☐ 6-9 months
    - ☐ 1 year or more

18. **If you are using the new classification system, how long does it take you on average to classify periodontitis in patients? (Please tick one)**
    - ☐ ≤3minutes
    - ☐ 4-5 minutes
    - ☐ 6-9 minutes
    - ☐ 10-14 minutes
    - ☐ 15-19 minutes

PART 2

This section aims to evaluate current understanding of the ***<u>new 2017 World Workshop Periodontology</u>*** *classification of Periodontal diseases.*

1. **According to the 2017 World Workshop periodontal classification, a patient has generalised disease when how many teeth are affected? (Please tick one)**
   - ☐ <30% of sites affected
   - ☐ >30% of sites affected
   - ☐ <30% of teeth affected
   - ☐ >30% of teeth affected
   - ☐ Only molars and incisors affected
   - ☐ Unsure/Do not know

2. **According to the 2017 World Workshop periodontal classification, the stage is primarily determined based on: (Please tick one or more)**
   - ☐ Clinical attachment loss at the worst affected site
   - ☐ Bone loss at the worst affected site
   - ☐ Pocket depths at the worst affected site
   - ☐ All of the above
   - ☐ None of the above
   - ☐ Unsure/Do not know

3. **According to the 2017 World Workshop periodontal classification, what parameters do you use in order to grade periodontitis for a patient who presents for the first time? You have no previous records/x-rays. (Please tick one or more)**
   - ☐ Clinical Attachment
   - ☐ Bone loss
   - ☐ Tooth loss
   - ☐ Age
   - ☐ Bone loss in relation to age
   - ☐ Smoking
   - ☐ Diabetes
   - ☐ Unsure/Do not know

4. **CASE 1:**
   Based upon the X-ray below, what would be the stage of periodontitis for this case using the World Workshop of Periodontology 2017 classification.

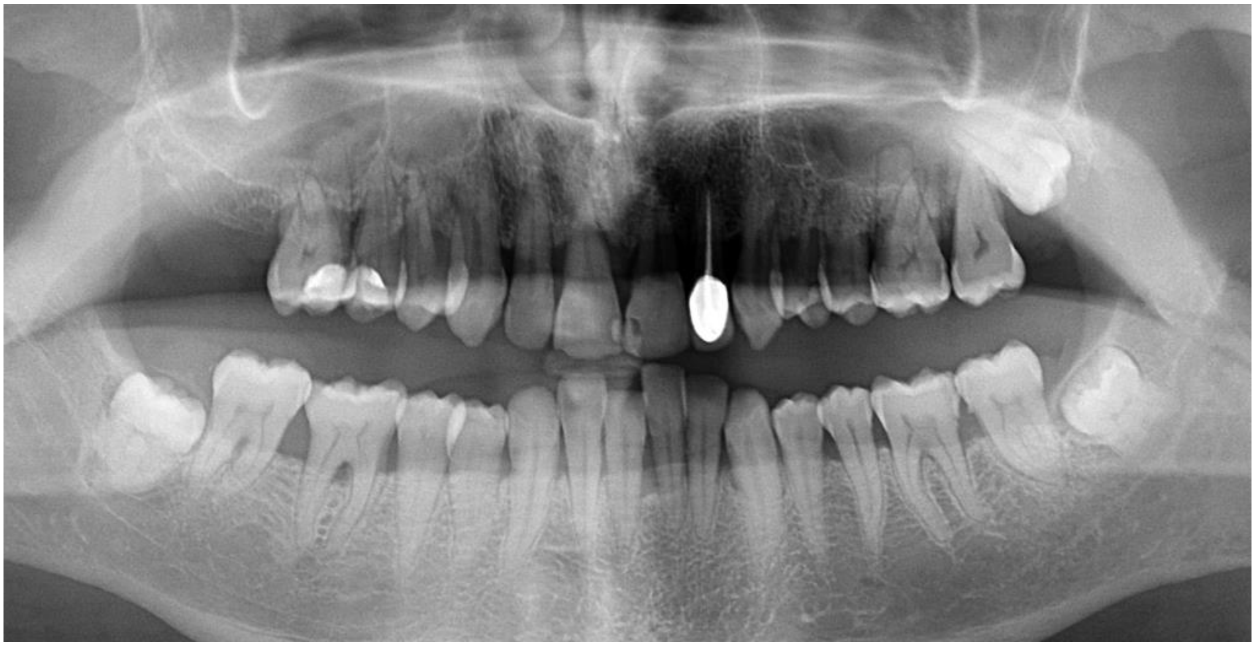

- ☐ Stage 1
- ☐ Stage 2
- ☐ Stage 3
- ☐ Stage 4
- ☐ Unsure/Do not Know
- ☐ Other …………………….

5. **CASE 2:**
   A 45-year-old male patient attends for the first time at your practice. Upon clinical and radiographic examination the worst affected site is LL6. Using the X-ray below and the 2017 WWP classification, what is the grading of this case?

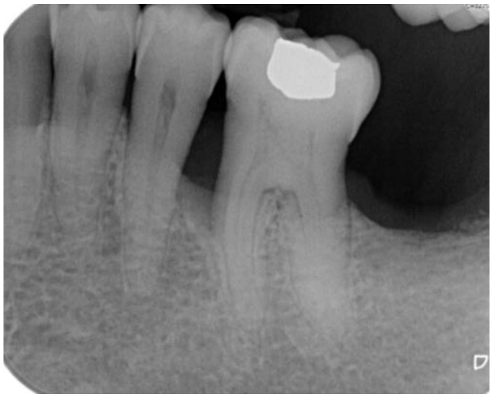

- ☐ Grade A
- ☐ Grade B
- ☐ Grade C

- ☐ Unsure/Do not know
- ☐ Other ………………………………..

PART 3

This section aims to evaluate potential areas for future development

1. **Do you think that you need to improve your knowledge and understanding about the new classification? (Please tick one)**
   - ☐ Yes
   - ☐ No
   - ☐ Unsure

2. **If yes, how will you like to increase your knowledge and understanding (Please tick one or more)**
   - ☐ Continual Professional Development
   - ☐ Journals
   - ☐ Peer discussions
   - ☐ Webinars
   - ☐ Lectures/Seminars
   - ☐ Other (please specify)…………………………………….

Thank you for participating in this questionnaire project. Your co-operation is greatly appreciated.

Εργαστήριο Προληπτικής Οδοντιατρικής, Περιοδοντολογίας και Βιολογίας Εμφυτευμάτων, Α.Π.Θ.

Barts and The London School of Medicine and Dentistry
